# COVID-19 vaccination status among people with selected citizenships: results of the study GEDA Fokus

**DOI:** 10.25646/11142

**Published:** 2023-03-21

**Authors:** Marleen Bug, Miriam Blume, Katja Kajikhina, Susanne Bartig, Elisa Wulkotte, Hannelore Neuhauser, Julia Geerlings, Claudia Hövener, Carmen Koschollek

**Affiliations:** 1 Robert Koch Institute, Berlin Department of Epidemiology and Health Monitoring; 2 Robert Koch Institute, Berlin Department of Infectious Disease Epidemiology

**Keywords:** MIGRATION, EDUCATION, COVID-19, VACCINATION, HEALTH INEQUALITY

## Abstract

**Background:**

the COVID-19 vaccination offers protection against severe disease progression. Data show that people with a history of migration are less likely to be vaccinated against COVID-19 than people without a history of migration, but are at increased risk of infection.

**Methods:**

Data were used from the GEDA Fokus interview survey (November 2021 – May 2022), which included people living in Germany with Croatian, Italian, Polish, Syrian or Turkish citizenship (n=5,495). In addition to bivariate analyses, Poisson regressions were used to examine the association between uptake of at least one COVID-19 vaccination and sociodemographic, health- and migration-related factors.

**Results:**

90.0% of participants reported having received at least one COVID-19 vaccination. Having visited a general practitioner or specialist in the past 12 months, living in Germany for 31 years or more, and having a greater sense of belonging to society in Germany were associated with vaccination uptake in bivariate analyses. Regression analysis showed that older people and those with higher education were more likely to be vaccinated.

**Conclusions:**

Sociodemographic factors are associated with uptake of the COVID-19 vaccine among individuals with selected citizenships. Low-threshold information and vaccination offers are important to ensure equal access to vaccination.

## 1. Introduction

At the end of 2019, the novel coronavirus SARS-CoV-2, which can cause COVID-19 disease, was discovered for the first time. Due to high infection rates by droplet infection, a pandemic situation was quickly reached with severe health and social consequences [1]. Vaccination is considered a key tool in the fight against infectious diseases. They help to reduce the rate of new infections by providing varying degrees of immunity (e.g., protection against infection, protection against disease, protection against severe disease progression) [2]. With the start of the vaccination campaign in Germany at the end of December 2020, a major step has been taken to contain the COVID-19 pandemic in this country [3]. However, the ability and willingness to be vaccinated against COVID-19 depends on a number of factors. In addition to fears, doubts, and a perceived low risk of one’s own exposure to the virus [4, 5], barrier-free access to information and vaccination services is significantly associated with vaccine uptake [6]. In addition, recent studies show that COVID-19 vaccination uptake is associated with a person’s socioeconomic position, and people with lower socioeconomic position tend to have lower COVID-19 vaccination coverage [7–10].

**Info box**

**History of migration, migration background – what terms do we use to describe what?**

People with a migration background or history of migration, immigrants and their (direct) descendants, people with an international history – various terms have been used in recent years to speak about migration and about people living in Germany. In this article, we use the term ‘people with a history of migration’ to refer to people who have immigrated themselves or whose parents have immigrated; however, this term is not intended to replace the statistical category of ‘migration background’.

The concept of ‘migration background’ has been increasingly criticised for multiple reasons, for example, by migrant self-organisations or by the Federal Expert Commission on the Framework Conditions for Integration Capability [37]. Therefore, we suggest that the concept should no longer be applied. On the one hand, the ‘migration background’ is often operationalised in studies in the health sciences differently than in the official statistics. Studies often conflate country of birth and current citizenship [38–40], whereas the definition of the Federal Statistical Office refers to one’s own and/or parental citizenship at birth [41]. In the general public, the term is often applied without a clear definition and serves to describe people who are German but are supposedly perceived as ‘not from here’. Since its introduction, the term has also experienced a development towards a stigmatising attribution to others [42] and is now mostly rejected as a self-description.

In contrast, the term ‘people with a history of migration’ is often used as a self-description of people who immigrated themselves or whose families have a biographical reference to migration or flight. Again, this term describes a very heterogeneous group of people. Therefore, rather than using aggregate categories such as ‘migration background’ or ‘history of migration’, we recommend analyzing relevant migration-related single indicators combined with other social determinants of health, depending on the particular research question for a differentiated analysis of migration and health. This approach is essential for making differentiated conclusions about factors and explanatory mechanisms of health inequalities.

Although there are no comprehensive studies for Germany so far, some studies suggest that people with a history of migration have lower COVID-19 vaccination rates and higher COVID-19 mortality [10–14]. At the same time, recent studies from the US and UK show that people with a history of migration have an increased risk of COVID-19 infection and an increased risk of severe disease progression [11, 15–19]. An increased risk of infection can be the result of different living conditions. Mechanisms of social exclusion, for instance, shape housing (e.g., communal accommodation, cramped living space) [20] and working conditions (e.g. inadequate infection and health protection in the workplace with increased exposure) [21–23]. People with a history of migration are more likely to work in systematically important occupations, such as retail or health care [24]. These circumstances can significantly increase the risk of an infection, as these occupations typically involve close contact with customers or patients, a lack of ‘physical distancing’, and exclude working from home during the pandemic [25]. Residence status or duration of residence can also be associated with the risk of an infection, as well as the risk of a severe disease progression. These factors directly determine access to the health care system and utilization of health services, but they also have an indirect effect because they are linked to a person’s social, housing, and employment situation [26, 27]. However, severe disease progression might also be caused by preexisting health risk factors and conditions. These include, for example, diabetes mellitus and obesity [28]. Data from the general population in Germany show that these risk factors are more prevalent in socioeconomically disadvantaged groups [29]. People with a history of migration have, on average, a lower socioeconomic status and are more often affected by educational disadvantage and (the risk of) poverty [30]. These factors also influence health, especially in relation to (standard) health care and other resources, such as social and cultural capital or health-promoting living conditions [21, 31].

When describing the health situation of people with a history of migration, it is important to note that the term is used to describe an extremely heterogeneous group of people who need to be considered in a differentiated way and whose needs and resources are shaped by a variety of social determinants [32]. For example, people with a history of migration differ in their motives for moving to Germany, as well as in the duration of their residence, possible experiences of discrimination, and their German language skills. Each of these factors can have a different impact on individual health and the utilization of health services, such as the COVID-19 vaccination [21, 32–34] (see Recommendations for collecting and analysing migration-related determinants in public health research).

While factors that promote and inhibit the uptake of COVID-19 vaccination in the general population in Germany have been studied regularly [12, 35, 36], data on people with a history of migration is currently still incomplete. In order to better reflect the heterogeneity of people with a history of migration, this article takes a differentiated look at the uptake of the COVID-19 vaccination and its determinants. The aim is to examine the relationship between COVID-19 vaccination uptake and sociodemographic, health- and migration-related factors among people with a Croatian, Italian, Polish, Syrian, and Turkish citizenship.

## 2. Method

###  

#### Sample design and study implementation

‘German Health Update: Fokus (GEDA Fokus)’ is a multilingual survey of people with selected citizenships (Croatian, Italian, Polish, Syrian, and Turkish), that was conducted as part of the project ‘Improving Health Monitoring in Migrant Populations’ (IMIRA II) at the Robert Koch Institute (RKI). The study aimed to collect comprehensive information on health status, health behaviour, living conditions, and the utilisation of health services, as well as to enable differentiated statements according to sociodemographic and migration-related characteristics [43]. The (core) indicators developed within the framework of IMIRA I to describe the health situation of people with a migration background formed a thematic focus of the survey content [33]. In addition, relevant migration-sensitive concepts for health monitoring were taken into account, such as subjectively perceived or self-reported discrimination or the sense of belonging to society in Germany [44]. Questions on COVID-19 infection and vaccination status were also collected.

Based on a sample of residents’ registration offices, participants were randomly selected from 99 cities and municipalities throughout Germany according to the characteristics of citizenship (1st, 2nd, or 3rd citizenship; accordingly, persons with dual citizenship are included). The selection of five citizenships (population) was based on model calculations using the foreigner statistics [45] and register movements [46] of the Federal Statistical Office from 2015–2017. Thus, the size of the citizenship groups, as well as the dynamics of people (inward and outward migration), were taken into account [43]. Thus, our study population included persons between 18 and 79 years of age with Croatian, Italian, Polish, Syrian, or Turkish citizenship who had their main residence in one of the selected cities and municipalities at the time of the data collection [43]. Persons for whom a conditional blocking notice under § 52 of the Federal Registration Act is deposited in the population register and who are accordingly registered as residing in institutions (e.g., collective accommodation centres for refugees) were included in the sampling.

The data collection was carried out sequentially in a mixed-mode design from November 2021 to May 2022. In addition to a multilingual web-based questionnaire, the participants could participate via a printed paper questionnaire in German or one of the five study languages (Arabic, Croatian, Italian, Polish, or Turkish). If there was no response, there was the possibility of a personal interview with partly multilingual interviewers or, in the larger cities, a telephone interview in the preferred language of the participant [43].

A total of 6,038 people (2,983 women and 3,055 men) participated in GEDA Fokus. The response rate was 18.4% (Response Rate 1), according to the standards of the American Association for Public Opinion Research (AAPOR) [47]. The study design of GEDA Fokus is described in detail in another article [43].

#### Outcome and determinants

The outcome variable uptake of COVID-19 vaccination (at least once) was collected using the question ‘Have you already been vaccinated against the coronavirus disease (COVID-19)?’. The response options were ‘Yes’, ‘No’, and ‘I do not want to answer this question’ and were dichotomised into ‘Yes’ vs. ‘No’. Respondents indicating ‘I do not want to answer this question’ were excluded from the present analysis (n=242).

The analyses only included people whose gender as reported in the population register matched the gender stated on their birth certificate (according to self-reports in the questionnaire). The age of respondents was categorised into the following groups: 18 to 39 years, 40 to 59 years, and 60 to 79 years. Education was categorised into low (ISCED 1–2), medium (ISCED 3–4), and high (ISCED 5–8) groups based on the educational and vocational qualifications of the study participants, according to the 2011 version of the International Standard Classification of Education (ISCED 2011) [48].

The indicators on primary medical or specialist health care were collected via two questions: 1) ‘When did you last consult a general practitioner or family doctor for advice, examination, or treatment?’ and 2) ‘When did you last consult a specialist for advice, examination, or treatment?’. The response options were: ‘less than 6 months ago’, ‘6 to less than 12 months ago’, ‘12 months ago or longer’, and ‘Never’. For the variable of specialist health care, the answers were dichotomised (‘less than 12 months ago’ vs. ‘more than 12 months ago/never’) due partly to the very small number of cases.

As a migration-related characteristic, the duration of residence was categorised into ‘since birth’, ‘up to and including 10 years’, ‘11 to 30 years’, and ‘31 years or more’. The current residence status was operationalised using the following characteristics: ‘German citizenship’, ‘EU citizenship’, ‘permanent residence status’, and ‘temporary residence status’. To map German language proficiency, the responses on mother tongue (‘German’, ‘another language’) and the self-assessed German language skills of those who did not state German as their mother tongue were used and combined into the following categories: ‘mother tongue, very good’, ‘good, moderate’ and ‘poor, very poor’.

The questionnaire asked about the frequency of reported experiences of discrimination (‘How often have you been treated unfairly or worse than other people in such a way in the following situations?’) ‘in the health or care sector (e.g., doctor, hospital, assisted living, care facility)’ [44]. Answers were categorised for the evaluations into ‘very often, often’, ‘sometimes’, and ‘rarely, never’. The sense of belonging to the society in Germany (‘How much do you feel you belong to the society in Germany?’) [44] was categorised into ‘very strongly, strongly’, ‘partly’, and ‘barely, not at all, does not apply’.

#### Data analysis

Cases with at least one missing value for one of the variables examined were excluded from the analyses (n=543), resulting in a final analysis sample of 5,495 participants. A weighting factor was included in the analysis to align the sample with the population of corresponding citizenships using the following characteristics: region, gender, age, education (ISCED 2011), and duration of residence [49]. These marginal distributions were taken from the 2018 Microcensus [50] after narrowing the data to the selected five citizenship groups (including dual citizenship). In order to adequately account for the clustering of participants within study locations and weighting when calculating confidence intervals and p-values, survey procedures for complex samples were used in all analyses [43, 51].

In the present article, the prevalence of at least one uptake of the COVID-19 vaccination is reported according to sociodemographic, health-related, and migration-related characteristics with 95% confidence intervals (95% CI). A significant difference is assumed if the p-value determined from the respective chi-square test is less than 0.05. In the following, only the results that are statistically significant according to the chi-square test are reported from the descriptive analyses, except for gender.

To complement the descriptive analyses, p-values were calculated from Poisson regressions to identify relevant associations with the uptake of COVID-19 vaccination. The regression analyses were adjusted for citizenship by registration offices. However, we refrain from reporting results by individual citizenship groups because, on the one hand, the sample composition probably differs systematically between the individual groups; therefore, comparability of these is difficult. On the other hand, the comparison runs the risk of being sweeping and stereotyped when describing individual effects according to citizenship.

In the multivariate analysis, all determinants were included that had a significant influence on the uptake of COVID-19 vaccination in the bivariate analyses.

## 3. Results

###  

#### Sample description

Among the 5,495 participants included in the analyses, slightly more were men (54.5%) than women (45.5%) (Table 1). Most participants belonged to the lower (43.9%) or middle (41.2%) education group. More than three-quarters of the participants (77.9%) had seen a general practitioner less than 12 months previously, and just over half (53.6%) had visited a specialist in the same period. In terms of migration-related factors, the most commonly reported duration of residence was 31 years or more (28.8%), and the most commonly reported residence status was EU citizenship (40.5%). Almost half (46.6%) of the participants rated their knowledge of German as good or moderate. A similar number (46.5%) reported having a native or very good level of German. The majority (86.1%) of respondents reported that they rarely or never experienced discrimination in the health or care sector; 3.8% experienced discrimination (very) often in this context. Almost two-thirds (64.4%) of respondents reported a (very) strong sense of belonging to the society of Germany (Table 1).

### Determinants of COVID-19 vaccination: bivariate analyses

#### Sociodemographic determinants

**Figure 1:**
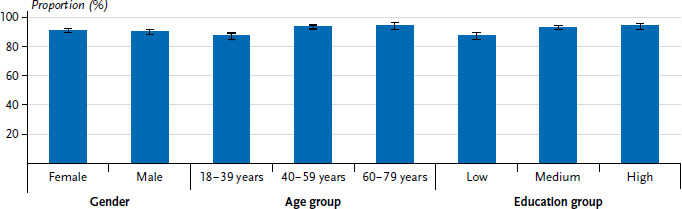
Prevalence (%) of at least one COVID-19 vaccination uptake by sociodemographic characteristics (n=2,704 women and n=2,791 men) Source: GEDA Fokus (2021–2022)

Of all participants, 90.0% reported having been vaccinated against COVID-19 at least once (Table 1), although the proportion was slightly lower among men (89.6%, 95% CI: 87.4–91.4%) than among women (90.5%, 95% CI: 88.4–92.3%, p=0.5012) (Figure 1). The proportion of people vaccinated against COVID-19 increases with age. While 86.6% (95% CI: 83.9–88.9%, p<0.001) of 18- to 39-year-olds reported having been vaccinated, the figure was 93.9% (95% CI: 90.4–96.1%, p<0.001) for respondents aged 60 to 79. The prevalence of having received the COVID-19 vaccine at least once also varied by educational status. Respondents in the lower education group (86.7%, 95% CI: 83.8–89.2%, p<0.001) were almost 7 percentage points less likely to report having been vaccinated than those from the higher education group (93.4%, 95% CI: 90.4–95.6%, p<0.001). The prevalence of COVID-19 vaccination, which varies according to education status (high education group), differs little between female and male respondents (93.1% vs. 93.7%).

#### Health-related determinants

**Figure 2:**
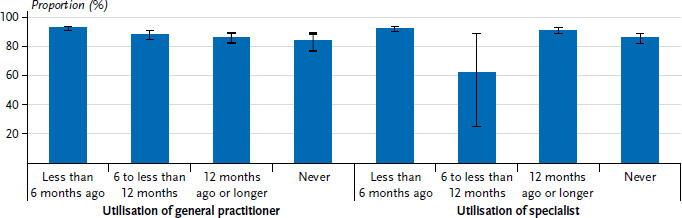
Prevalence (%) of at least one COVID-19 vaccination uptake by health-related characteristics (n=2,704 women and n=2,791 men) Source: GEDA Fokus (2021–2022)

In addition to sociodemographic characteristics, the prevalence of COVID-19 vaccination also varied by health-related characteristics (Figure 2). Participants who had visited a general practice in the last 6 months were more likely to report having been vaccinated (92.2%, 95% CI: 90.4–93.8%, p<0.001) than those who had visited primary care 12 months ago (85.7%, 95% CI: 81.8–89.0%) or never (83.4%, 95% CI: 76.0–88.8%, p<0.001). A similar gradient is also seen in the utilisation of specialist care. Study participants who had visited a specialist in the last 12 months (91.3%, 95% CI: 89.2–93.1%, p=0.0404) had a COVID-19 vaccination prevalence almost 3 percentage points higher than those who had never visited a specialist practice (88.4%, 95% CI: 86.2–90.3%). No gender differences were found in either primary care or specialist care.

#### Migration-related determinants

**Figure 3:**
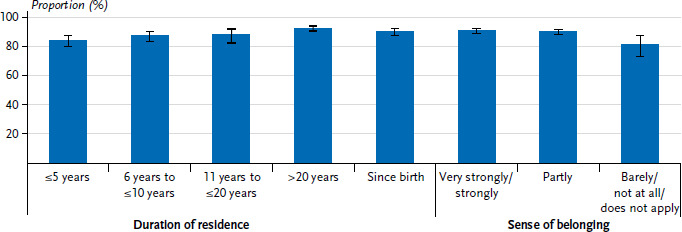
Prevalence (%) of at least one COVID-19 vaccination uptake by migration-related characteristics (n=2,704 women and n=2,791 men) Source: GEDA Fokus (2021–2022)

The prevalence of COVID-19 vaccination also varied according to migration-related characteristics (Figure 3). For example, study participants who had been in Germany for 31 or more years (92.2%, 95% CI: 89.5–94.3%, p=0.0240) were most likely to report having been vaccinated, compared with those who had been in Germany for less than ten years (86.3%, 95% CI: 82.9–89.3%, p=0.0240). There were no differences in prevalence between the gender. It is also shown that sense of belonging to society in Germany is associated with vaccination uptake. People who feel a strong or very strong sense of belonging to German society (90.9%, 95% CI: 88.7–92.7%, p=0.0101) are more likely to be vaccinated than those who feel partly (90.1%, 95% CI: 87.6–92.2%, p=0.0101) or barely/not at all (81.6%, 95% CI: 72.8–88.1%, p=0.0101). Gender differences are particularly pronounced in the group who feel that they barely or not at all belong to society in Germany. Compared to male respondents, women show a much higher prevalence of COVID-19 vaccination (77.8% versus 86.5%).

With regard to migration-related determinants of residence status, self-assessed German language skills and self-reported discrimination in the health and care sector, the bivariate analyses showed no significant differences within the groups.

#### Determinants of COVID-19 vaccination: multivariate Poisson regression analyses

The Poisson regression model shows that, regarding sociodemographic determinants, a higher age (40 years or older), as well as a medium or high education group, are positively associated with COVID-19 vaccination uptake (Table 2). Among the health-related determinants, only the utilization of primary medical care showed a significant positive association in the Poisson regression. In addition to specialist health care, none of the migration-related determinants showed a significant association with COVID-19 vaccination when examined in the regression model.

## 4. Discussion

This paper examines possible associations between sociodemographic, health-related, and migration-related factors, and COVID-19 vaccination uptake among people with Croatian, Italian, Polish, Syrian, or Turkish citizenship. 90.0% of respondents reported having received at least one COVID-19 vaccination. This proportion is much higher than in the general population in Germany (77.9%) [52]. Differences may be due to selection bias regarding willingness to participate. It is conceivable that people who are generally critical of the COVID-19 vaccination are less likely to have participated in GEDA Fokus. However, there are studies showing that people with a history of migration have a lower COVID-19 vaccination rate than people without a history of migration (12). The high vaccination rate in the context of the present analysis may be explained by the composition of the sample (Table 1). Participants very often reported good to very good German language proficiency and rarely if ever reported having experienced discrimination in the health or care sector. Also, in terms of residence status, a large proportion of the participants were EU or German citizens, and therefore experienced fewer barriers to accessing and using health services. Differences in vaccination behaviour are sometimes more likely to be explained by socioeconomic and sociodemographic factors (36, 54).

Age of 40 years or older, a higher education, and the use of primary health care in the last 12 months were positively associated with the uptake of COVID-19 vaccination. Specialist health care, duration of residence, and sense of belonging to society in Germany had a significant bivariate influence on vaccination behaviour but were no longer significant in the Poisson regression model. Self-assessed German language proficiency and self-reported experience of discrimination in the health or care sector were not significantly associated with COVID-19 vaccination in any of the analyses.

###  

#### Associations between sociodemographic factors and COVID-19 vaccination status

The relationship between age and uptake of COVID-19 vaccine is already known from studies in the general population [53]. Elderly people have a special indication for vaccination as they belong to a COVID-19 risk group [54, 55]. As in studies without a focus on migration, our results on people with selected citizenships show that more highly educated people are more likely to be vaccinated [56, 57]. The reasons for the correlation between educational level and vaccination behaviour are complex and not migration-specific. For example, higher education is often beneficial for the health literacy [58–60]. This refers to the ability and willingness to find prevention and care services, to overcome potential problems of understanding and application, and to navigate oneself in the care structures. Greater health literacy may therefore have a positive influence on vaccination behaviour. The results of the article thus confirm the existing findings on the general population, also in a sample of people with selected citizenships.

#### Associations between health-related factors and COVID-19 vaccination status

Our results show that respondents who had received primary health care in the past 12 months were also more likely to have received at least one vaccination. It is possible that respondents consulted a general practitioner for the COVID-19 vaccine, although it is likely that large parts of the population received the vaccine in other settings, such as vaccination centres. This is because many people avoided doctors’ offices during the first months of the pandemic, presumably out of fear of infection [61]. Nevertheless, the vaccination could have been offered and taken up during an otherwise justified visit to the medical practice. A study from the US on COVID-19 vaccination intentions shows that for a large part of the respondents, medical staff is considered the most trustworthy source of COVID-19 information [62]. There is evidence that people with a history of migration are less likely to utilise primary health care services than people without a history of migration [63], or at least certain subgroups among them, such as those with a shorter duration of residence or temporary residence status [64]. Recommendations for vaccination by health professionals may have reached people more easily who visit doctors at shorter intervals [65]. In addition, the willingness to accept recommended treatments is higher if a general practice has been visited [66]. Therefore, it seems advisable to remove barriers to the utilisation of general medical services and to facilitate access to them. In addition to a reduced trust in the health care system, a lack of orientation and health literacy and communicative barriers, such as a lack of language mediation or an improvement in the relationship between doctors and their patients, can also play an important role [59, 67, 68].

#### Associations between migration-related factors and COVID-19 vaccination status

Our analyses show bivariate associations between individual migration-related factors and the uptake of COVID-19 vaccination. Factors that were positively associated with vaccination uptake were a longer duration of residence of 31 years or more and a strong sense of belonging to the society in Germany. As expected, a longer duration of residence is associated with increasing age. Given that older people are considered a COVID-19 risk group, and thus have a special vaccination indication as described [54], the association between duration of residence and vaccination status could be explained. Similarly, longer duration of residence is often associated with improved language skills, which helps to overcome barriers in access to health care. [69]. Particularly in the COVID-19 information gathering and vaccination decision-making, a longer duration of residence could be conducive to vaccination readiness.

Furthermore, the bivariate analyses showed a correlation between a strong sense of belonging to society in Germany and more frequent self-reported COVID-19 vaccination. This aspect is also supported by previous research literature and there are data reporting an influence of psychological determinants on willingness to vaccinate [4]. For example, the existence of a social sense of community can be essential to the decision for or against vaccination. When age and education were taken into account, a statistically significant correlation between the COVID-19 vaccination and the duration of residence, as well as the sense of belonging to society in Germany, was no longer found in the multivariate analysis. Thus, age and education seem to be much more relevant for vaccination uptake than individual migration-related factors.

Language skills are often cited as a key factor in access to health care and utilisation of health services [70, 71]. In terms of vaccination behaviour in the COVID-19 pandemic, better German language skills also seem to be associated with increased willingness to vaccinate [12]. The present paper could not confirm this correlation. One possible reason for this could be the composition of the sample studied. For example, respondents often reported a longer duration of residence in Germany and predominantly mother-tongue or very good knowledge of German.

Similarly, the correlations between health and vaccination behaviour and experiences of discrimination that are often described in the literature could not be confirmed in the context of these analyses [21, 72, 73]. It is therefore possible that there is a bias in the sample, resulting in an insufficient number of cases of people with poor German language skills or people who have experienced discrimination, who may have decided not to participate in the study. Due to the currently rather limited number of studies on the connection between vaccination behaviour and language skills, as well as experiences of discrimination, this potential relationship should be investigated in further studies.

#### Strengths and limitations

The data collected in the Survey GEDA Fokus provide the opportunity to look at various socio-demographic, health-related and migration-related factors in a differentiated way, based on a large sample of people with selected citizenships from all over Germany. The use of various diversity-and migration-sensitive measures, such as translated study materials and multilingual interviewers, can be described as profitable in terms of response rates. All concepts, such as self-reported discrimination or the sense of belonging to society in Germany, were cognitively tested in advance as part of a feasibility study in the IMIRA project and thus adapted for the GEDA Fokus survey study. One limitation lies in the selection of the sample. The sample was drawn solely on the basis of citizenship. This meant that large subgroups among people with a history of migration, such as naturalised citizens, were excluded from the survey. The same applies to people with citizenships other than the five selected. People who were not registered at the residents’ registration offices, but who nevertheless resided permanently in Germany, could not be included in the study either. Even though the five selected citizenship groups comprise a large part of the group of people with a history of migration living in Germany, it is not possible to draw general conclusions about this group.

Although the possibility of participating in the survey in six different languages led to an increased willingness to participate and thus appeared to be purposeful, the limitation to the five translation languages Arabic, Croatian, Italian, Polish, and Turkish may represent a further limitation. Other translations, for example into Kurdish for people with Turkish or Syrian citizenship, could not be realised due to limited time and financial resources. Another limitation is the use of different modes in cities of different sizes. For example, the personal interview was only possible in selected locations and therefore only for some of the study participants. In addition, the response rate of 18.4% is lower than in the GEDA 2014/2015-EHIS study of 26.9% [74], which was also based on a residents’ registration office sample, but targeted the general population. However, the sequential design with several modes of participation in different languages favoured the inclusion of different subgroups, so that a possible bias in the willingness to participate could be well countered [75]. Particularly with regard to the subject matter of COVID-19 and the reference of the study to the RKI, there is the possibility of a bias in participation due to the sensitivity of the topic, so that people who are rather sceptical about the topic – and thus also about vaccination itself – may have participated less frequently. The responses themselves could also be biased by social desirability. This effect may be particularly strong in face-to-face interviews [76]. The data do not confirm this, at least for the question on COVID-19 vaccination, as respondents in personal interviews were more likely to report that they had not been vaccinated. It is possible that the number of unvaccinated cases (n=516) was still too small for the analyses to show significant correlations across many subgroups.

#### Conclusion

This paper provides valuable insights into the factors influencing COVID-19 vaccination among people with Croatian, Italian, Polish, Syrian, or Turkish citizenship. It has been shown that, when considering this specific population group, as in the general population, it is primarily sociodemographic and less migration-related factors that determine the COVID-19 vaccination uptake. The level of education is particularly important for the COVID-19 vaccination rate. Furthermore, the results reflect the heterogeneity of the lives of people with a history of migration, also with regard to the COVID-19 vaccination (see Recommendations for collecting and analysing migration-related determinants in public health research). In practice and in relation to the COVID-19 pandemic, the results mean that target group-specific prevention and infection protection measures should be ensured, such as low-threshold, lifeworld-related, and free vaccination and information services. The last mentioned should be migration-sensitive and multilingual [8] to improve the possibility of access. In addition, structural barriers to access to general medical and specialist health care services must be addressed and eliminated in the long term. To counteract the health inequalities exacerbated by the SARS-CoV-2 pandemic, the aim should be to ensure equal access to COVID-19 vaccination for all people.

## Key statement

- Previous studies have shown an increased risk of infection with COVID-19 among people with a history of migration, while showing lower vaccination rates.
- The GEDA Fokus survey allows a differentiated description of different factors influencing the COVID-19 vaccination status of people with selected citizenships.
- Utilisation of primary health care is positively associated with COVID-19 vaccination uptake.
- Increasing age and higher education are associated with COVID-19 vaccination uptake.
- Low-threshold information, counselling and vaccination services tailored to the target group are important for the uptake of COVID-19 vaccination.

## Figures and Tables

**Table 1:** Sample description by sociodemographic, health- and migration-related factors (n=2,704 women and n=2,791 men) Source: GEDA Fokus (2021–2022)

Number of cases (n)		Weighted sample (%)
**Outcome**		
**COVID-19 vaccination (at least once)**		
Yes	4,997	90.0
No	498	10.0
**Sociodemographic factors**		
**Gender**		
Female	2,704	45.5
Male	2,791	54.5
**Age group**		
18 – 39 years	2,817	47.8
40 – 59 years	1,959	37.7
60 – 79 years	719	14.5
**Sociodemographic factors**		
**Education group**		
Low	1,479	43.9
Medium	2,082	41.2
High	1,934	14.9
Citizenship^*^		
Croatian	1,155	18.1
Italian	1,141	19.2
Polish	1,114	21.7
Syrian	1,121	15.3
Turkish	1,105	25.7
**Health-related factors**		
**Utilisation of general practitioner**		
Less than 6 months ago	3,347	61.6
6 to less than 12 months ago	912	16.3
12 months ago or longer	966	16.4
Never	270	5.6
**Utilisation of specialist practitioner**		
Less than 12 months ago	3,091	53.6
12 months ago or longer/Never	2,404	46.4
**Migration-related factors**		
**Duration of residence**		
≤10 years	2,261	27.6
11 years to ≤30 years	936	23.1
≥31 years	1,166	28.8
Since birth	1,132	20.5
**Residence status**		
German citizenship	1,479	28.6
EU citizen	2,327	40.5
Permanent residence status	750	17.7
Temporary residence status	939	13.3
**German language proficiency**		
Mother tongue/very good	2,436	46.5
Good/moderate	2,571	46.6
Poor/very poor	488	6.9
**Experience of discrimination**		
Very often/often	203	3.8
Sometimes	545	10.1
Rarely/never	4,747	86.1
**Sense of belonging**		
Very strongly/strongly	3,372	64.4
Partly	1,633	28.2
Barely/not at all/does not apply	490	7.5

% (weighted), n (unweighted),

^*^according to Residents’ Registration Offices

**Table 2:** Prevalence (%) of at least one COVID-19 vaccination uptake to sociodemographic, health-related, and migration-related factors; results of Poisson regression (n=5.495) Source: GEDA Fokus (2021–2022)

%		(95% CI)	PR	p-value
**Sociodemographic factors**				
**Gender**				
Female	90.5	(88.4-92.3)	Ref.	
Male	89.6	(87.4-91.4)	0.98	0.361
**Age group**				
18-39 years	86.6	(83.9-88.9)	Ref.	
40-59 years	92.8	(90.8-94.3)	1.07	**0.000**
60-79 years	93.9	(90.4-96.1)	1.10	**0.001**
**Education group**				
Low	86.7	(83.8-89.2)	Ref.	
Medium	92.2	(90.2-93.8)	1.08	**0.000**
High	93.4	(90.4-95.6)	1.10	**0.000**
**Health-related factors**				
**Utilisation of general practitioner**				
Less than 6 months ago	92.2	(90.4-93.8)	Ref.	
From 6 to less than 12 months ago	88.0	(84.2-91.0)	0.96	0.064
12 months ag or longer	85.7	(81.8-89.0)	0.94	**0.004**
Never	83.4	(76.0-88.8)	0.94	0.123
**Utilisation of specialist practitioner**				
Less than 12 months ago	91.3	(89.2-93.1)	Ref.	
12 months ago or longer/Never	88.4	(86.2-90.3)	1.00	0.882
**Migration-related factors**				
**Duration of residence**				
≤10 years	86.3	(82.9–89.1)	Ref.	
11 years to ≤30 years	91.2	(87.4–94.0)	1.04	0.310
≥31 years	92.2	(89.5–94.3)	1.02	0.662
Since birth	90.4	(87.0–93.1)	1.03	0.351
**Sense of belonging**				
Very strongly/strongly	90.9	(88.7–92.7)	Ref.	
Partly	90.1	(87.6–92.2)	1.00	0.815
Barely/not at all/does not apply	81.6	(72.8–88.1)	0.92	0.067

Adjusted for citizenship according to population registration office, PR=prevalence ratio, ISCED=International Standard Classification of Education 2011 (50), CI=confidence interval, Ref.=reference group
